# Management of Lateral Epicondylitis Using Transdermal Nitroglycerin: A Systematic Review

**DOI:** 10.7759/cureus.32560

**Published:** 2022-12-15

**Authors:** Cara McCulloch, Miles M Hunter, Chris Lipp, Eddy Lang, Heather Ganshorn, Pavneet Singh

**Affiliations:** 1 Cumming School of Medicine, University of Calgary, Calgary, CAN; 2 Department of Emergency Medicine, University of Calgary, Calgary, CAN; 3 Department of Innovative Sport Medicine, University of Calgary, Calgary, CAN

**Keywords:** tendinitis, tendinopathy, lateral epicondylitis, tennis elbow, emergency medicine, transdermal nitroglycerin, topical nitroglycerin, emergency medicine pain managementanaesthesia

## Abstract

Lateral epicondylitis (LE), also known as tennis elbow, is an overuse tendinopathy originating from the forearm extensor tendons of the elbow. An emerging therapy for the treatment of LE is the use of transdermal nitroglycerin (NTG) patches for pain relief and improved function. The aim of this systematic review was to assess the current literature on the effect of a transdermal NTG patch for the treatment of LE. A literature search using MEDLINE, EMBASE, SportDiscus, and the Cochrane Database of Systematic Reviews was conducted. Studies selected for inclusion were those in which patients were clinically diagnosed with LE, RCTs, observational studies, and only articles published in English. Studies were excluded if they involved patients <18 years of age or involved patients with a potential alternative source of elbow pain such as previous surgery to the elbow, a previous history of dislocation, fracture of the elbow or tendon rupture, or a referred pain source such as cervical radiculopathy or peripheral nerve involvement. Studies were also excluded if they involved patients who were already prescribed topical NTG for any other indication (i.e., angina), and if the studies had no measurement of symptom relief or measurement or functional scoring. The initial search strategy yielded 69 articles, out of which four met the eligibility criteria and were included in this systematic review. The studies showed improvement in elbow pain in the short-term and mid-term (up to six months), while one study that followed participants for a five-year duration post-treatment, showed no benefit. Three studies used an effective NTG dose of 1.25mg/24h and one study used an effective dose of 1.44mg/24h. Topical NTG was more effective when combined with a tendon rehabilitation program. The most commonly reported side effects of topical NTG were headaches and dermatitis. Overall, the current literature demonstrates that the use of NTG patches for LE improves short- and mid-term pain as well as elbow function. However, more studies are required to fully understand the effect of topical NTG on LE, particularly the effective dose range and the long-term benefits.

## Introduction and background

Lateral epicondylitis (LE), also known as tennis elbow, is an overuse tendinopathy originating from the forearm extensor tendons as they attach to the lateral epicondyle of the elbow. Despite the suffix “itis,” which suggests an inflammatory etiology, the pathology is no longer thought to be inflammatory in nature [1,2]. Although the pathophysiology of LE is not fully understood, it is now recognized as degenerative overuse tendinopathy (i.e., tendinosis) secondary to repetitive trauma to tendon fibrils [1,2]. Clinical features of tennis elbow include tenderness at the lateral epicondyle, and pain on resisted wrist extension or forearm supination, with normal elbow range of motion [3]. Mechanism of injury is commonly seen in sedentary individuals who transition between periods of underuse to overuse on a frequent basis, along with both recreational and high-level athletes [3]. LE can potentially occur in any sports or occupation that involves repetitive and prolonged gripping including golf, pickleball, climbing, motocross, mountain biking, gardening, painting, or carrying luggage for extended distances. Therefore, patients may present to the Emergency Departments, urgent care centers or primary care with LE secondary to any of the aforementioned activities. Traditional treatment regimens for LE have focused on the use of anti-inflammatory medications such as oral or topical non-steroidal anti-inflammatories (NSAIDs), and corticosteroid injections [1]. However, the absence of any associated inflammatory markers has led practitioners to question the use of these traditional treatment regimens [4]. Consequently, the management of LE has evolved in recent years with the mainstay of treatment being rest, ice, stretching, various braces or tension straps, and platelet-rich plasma injections, followed by a transition to rehabilitation programs with graduated loading exercises [3,4]. However, many of these treatment strategies can only be initiated after the resolution of acute pain from the injury. As such, multiple therapies have been trialed to shorten the acute phase of the tennis elbow and accelerate the rehabilitation process. Two particular therapies for treating LE in patients include corticosteroid injection and topical nitroglycerin (NTG) [3].

While corticosteroid injection has been shown to have promising effects in the short term when compared to sodium hyaluronate, botulinum toxin, prolotherapy, platelet-rich plasma and placebo, its recurrence rate is higher than physiotherapy alone, or even a simple wait-and-see approach [4]. As a result, the use of corticosteroid injection in the emergency department for the treatment of LE is more appropriate when immediate short-term relief is critical to the patient, despite the increased risk of recurrence [3].

A glyceryl trinitrate transdermal patch, more simply known as topical NTG, has been used to treat angina pectoris for over a hundred years. Its mechanism of action is through nitric oxide, an endothelial vasodilator, thought to also play a role in tendon healing [4,5] and has been shown to be effective in several tendinopathies [6]. Therefore, the aim of this systematic review was the evaluate the current literature on the effectiveness of NTG for treating LE in both the short term and long term. 

## Review

Materials and methods

The present systematic review was conducted and authored using guidelines from the Preferred Reporting Items for Systematic Reviews and Meta-Analyses (PRISMA) guidelines [7,8].

Search Strategy

A systematic search of the literature was performed using MEDLINE, Embase, SportDiscus, and the Cochrane Central Register of Controlled Trials from inception through to October 26th, 2020. We searched keywords and subject headings for the following concepts: tennis elbow and NTG. The complete MEDLINE strategy is included in Table 1.

**Table 1:** Search strategy. Ovid MEDLINE(R) and Epub Ahead of Print, In-Process & Other Non-Indexed Citations, Daily and Versions(R) 1946 to October 26, 2020.

#	Searches	Results
1	exp Tennis Elbow/	1683
2	(tennis elbow or acute lateral epicondylitis).kf,tw.	1061
3	1 or 2	2072
4	exp Nitroglycerin/	12300
5	(nitroglycerin* or glyceryl trinitrate or nitric oxide or Minitran or Nitro-Dur).kf,tw.	161668
6	4 or 5	165147
7	3 and 6	16

This search was translated to other bibliographic databases. A search was also performed using clinicaltrials.gov for studies recruiting or recently completed. An updated search was performed on December 7, 2022 with additional search terms for LE including lateral epicondylopathy, common extensor tendinopathy and common extensor tendinosis to identify any relevant articles published since the original search.

Eligibility Criteria

Studies were included if they involved patients who were clinically diagnosed with LE (as defined by pain over the lateral epicondyle, which is provoked by palpation, gripping, resisted wrist extension, or second or third finger extension) and were required to have a measurement system for either symptom relief or a functional outcome score compared to baseline; both RCTs and observational studies were included. Studies selected compared a treatment group with topical NTG compared to a control group who either received a placebo or who were prescribed rehab exercises. Only articles available in English were considered for inclusion in the final review. Studies were excluded if they involved patients <18 years of age or involved patients with a potential alternative source of elbow pain such as previous surgery to the elbow, a previous history of dislocation, fracture of the elbow or tendon rupture, or a referred pain source such as cervical radiculopathy or peripheral nerve involvement. Studies were also excluded if they involved patients who were already prescribed topical NTG for any other indication (i.e., angina), and if the studies had no measurement of symptom relief or measurement or functional scoring. 

Selection Process

**Figure 1:**
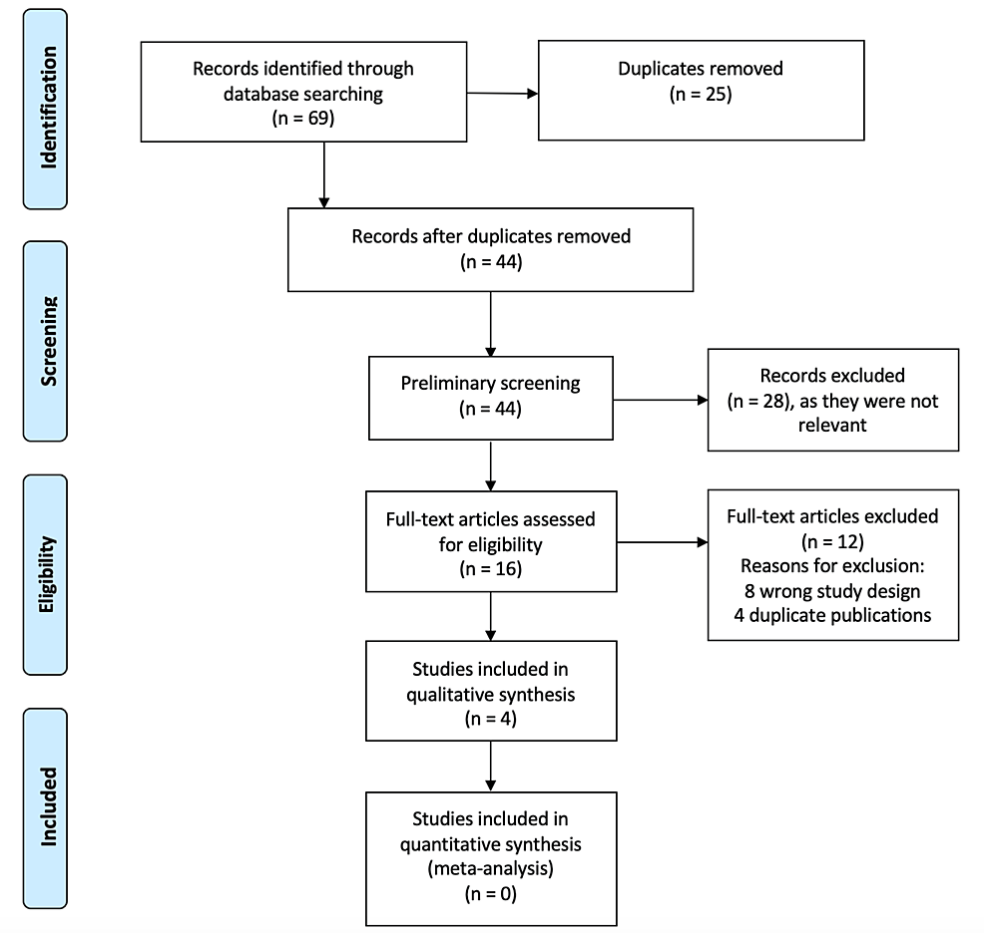
PRISMA diagram for article screening.

Article selection for inclusion was performed independently by two review authors (CM and CL) after the de-duplication of articles performed through Covidence software. Any disagreement over the eligibility of studies was resolved through discussion between the two review authors and a third author (MH). Figure 1 illustrates the article screening process according to PRISMA guidelines [7,8].

Data Collection

The four articles selected for inclusion were reviewed independently by authors CM and PS. Data were extracted using a standardized, pre-piloted form. Extracted data included information such as study setting, study population, participant demographics and baseline characteristics, details of the intervention and control conditions, study methodology, recruitment and study completion rates, primary and secondary outcomes, and study time points. Raw data were requested from study authors, although not provided due to lack of author response. The primary outcomes for which data was sought were a) a reduction in lateral epicondyle pain, both at rest and with activity, as measured by a visual analog scale (VAS); and b) measurement t of elbow function as defined by wrist extensor tendon mean peak force using the modified chair pick-up test (the Orthopaedic Research Institute-tennis elbow testing system; ORI-TETS), which is a previously validated scoring tool [9].

Secondary outcomes collected were side effect profiles or adverse reactions to the intervention. In cases where results were not published in numerical format, data was extracted manually from the published charts using Tabula software.

Risk of Bias

A thorough quality assessment of all the studies was conducted based on the Consolidated Standard of Reporting Trials (CONSORT) checklist to evaluate the quality of reporting for each study. The risk of the bias was assessed independently by two review authors (CM and MH) based on the Cochrane Risk of Bias tool, and late confirmed by a third author (PS). The studies were categorized as high, low, or unclear risk of bias based on the assessment.

Results

Search Outcomes

As identified in Figure 1 (PRISMA [Preferred Reporting Items for Systematic Reviews and Meta-Analyses] flow diagram), the search strategy yielded 69 records. After removing duplicates, 44 records were screened for eligibility using title and abstract to yield 16 articles. The full text of these articles was retrieved and screened for inclusion, rendering four studies that were included in this review. The study characteristics and results are highlighted in Table 2, with each study measuring outcomes at different time points. The updated search did not identify any new articles for inclusion.

**Table 2:** Study characteristics and results RCT = Randomized control trial, NTG = nitroglycerin

#	Authors	Country	Date	Journal	Treatment	Dose	Time Points Evaluated	Study Type	Participants	Attrition	Effect on Pain	Effect on Elbow Function	Side Effects
1	Palomi et al.	Australia	2003	The Americal Journal of Sports Medicine	Glyceryl trinitrate transdermal patch + tendon rehabilitation	1.25 mg/24 h	2-week, 6-week, 12-week, 6-month	RCT	n=86	n=12 (14%)	Reduced elbow pain with activity at 2 weeks (p=0.01), reduced epicondylar tenderness at 6 weeks (p=0.02) and 12 weeks (p=0.02), as assessed using a 4-point scale.	Increase in wrist extensor mean peak force and total work at 6-months (p=0.03). 81% of treatment group asymptomatic at 6-months vs 60% in control group (p=0.005). Mean estimated effect size at week 24 was 0.12 (95% confidence interval, 0.06 to 0.19).	Headaches, dermatitis rash, facial flushing, cutaneous angiodysplasia, ipsilateral axillary sweating, feeling of uneasiness/apprehension
2	Palomi et al.	Australia	2008	British Journal of Sports Medicine	Glycerl trinitrate patch + elbow rehabilitation	7.2 mg/24 h, 1.44 mg/24 h, and 3.6 mg/24 h	8-week	RCT	n=154	n=18 (11.7%)	Decrease in elbow pain with activity in the treatment group using 0.72mg/24 NTG compared to control group (p=0.04), using visual analog scale	No significant improvement in elbow function	Dose dependent headaches, dermatitis rash
3	McCallum et al.	Australia	2009	British Journal of Sports Medicine	Topical glycerl trinitrate patch + tendon rehabilitation	1.25 mg/24 h	6-month, 5-year	Prospective comparitive study	n=86	n=28 (32.6%)	No significant improvement in pain in treatment group vs control at 5-year follow-up using a 4-point scale	No significant improvement in elbow function in treatment group vs control at 5-year follow-up	Headaches and rash until 6-months. No sde effect reported for the 5-year follow-up
4	Ozden et al.	Tukey	2014	Acta Orthopaedica et Traumatologica Turcica	Topical glycerl trinitrate patch + tendon rehabilitation	1.25 mg/24 h	3-week, 6 months	RCT	n=40	n=0 (0%)	Lower visual analog scale score in treatment vs control group at 3-week and 6-months (3.15 vs 6.45 at 3-week; 0.70 vs 4.85 at 6-months; p=0.001)	Elbow function not evaluated in this study	Headache

Appraisal of the Studies

Using the Cochrane Risk of Bias tool, two studies were found to be at low risk of selection bias [10,11]. One study was high risk of selection bias [12] and one study had unclear selection bias [13]. Performance bias was low in three studies [10,11,14] and high in one study [12]. Detection bias was low in one study [11], high in one study [12] and unclear in two studies [10,13]. Attrition bias was found to be low in all four studies [10-13]. On the other hand, reporting bias was unclear in all four studies included in this systematic review [10-13]. The risk of bias assessment is summarized in Table 3. Data were not pooled because of heterogeneity in study methods and outcomes.

**Table 3:** Risk of bias assessment (L= low, H= high, U= unclear).

	Random Sequence generation (selection bias)	Allocation concealment (selection bias)	Blinding of participants and personnel (performance bias)	Blinding of outcome assessment (detection bias)	Incomplete outcome data (attrition bias)	Selective reporting (reporting bias)
Paoloni et al., 2003 [10]	L	L	L	U	L	U
Paoloni et al., 2009 [11]	L	L	L	L	L	U
McCallum et al., 2011 [12]	H	H	H	H	L	U
Ozden et al., 2014 [13]	U	U	L	U	L	U

Effectiveness of Transdermal NTG in Treating LE

The first study examining the role of topical NTG in treating LE was done by Paoloni et al. in 2003 [10]. The study was a prospective, randomized, double-blinded placebo-control clinical trial and included 86 participants. The treatment group received an active NTG transdermal patch (1.25mg/24h), while the control group received a placebo patch. Both groups underwent a standard tendon rehabilitation program which included rest from aggravating activities in the early stages, a muscle strengthening program, and regular stretching of the extensor carpi radialis brevis muscle and tendon. The study found that the treatment group had significantly reduced elbow pain with activity at two weeks, reduced epicondylar tenderness at six and 12 weeks, and an increase in wrist extensor mean peak force and total work at 24 weeks. At six months, 81% of treated patients were asymptomatic during activities of daily living, compared with 60% of patients who had tendon rehabilitation alone. The side effects in the treatment group included headaches, dermatitis rash, facial flushing, cutaneous angiodysplasia, ipsilateral axillary sweating, and a feeling of uneasiness/apprehension. In the control group, side effects included headache and dermatitis rash. However, there was no significant difference in the number of days affected by headaches or of the amount of paracetamol required for headaches between the two groups. Twelve participants dropped out from the study for various reasons (five from the treatment group due to side effects, three from the treatment group for non-treatment related reasons, and four from the control group due to non-study related reasons).

Paoloni et al. [11] did another study in 2009 which was an RCT with 154 participants, 136 of which completing the study in its entirety. Participants were divided into a treatment group and a control group, which received either eight weeks of NTG or a placebo patch, respectively. Both groups also underwent an elbow rehabilitation program which comprised of isolated contraction of the wrist extensor muscles for 10 seconds, 2 seconds of muscle relaxation, followed by wrist extensor stretching for 15-20 seconds. The NTG dosage ranged from 7.2mg/24h, 1.44mg/24h, and 3.6mg/24h. Outcome measures included subjective global assessment of change in elbow symptoms, patient-rated tennis elbow evaluation, visual analog pain at rest, visual analog pain with activity, visual analog pain intensity, grip strength, and strength testing. The authors found that there was a significant decrease in elbow pain with activity in the treatment group using 0.72mg/24h NTG compared to control group (p=0.04). However, no significant differences were found in treatment groups using higher dosages of 1.44mg/24h or 3.6mg/24h. The authors also found no statistical differences in other outcome measures such as pain at rest, pain at night, pain-free grip strength, maximum force, and subjective global assessment of change. Dose-dependent headaches and dermatitis rash were side effects noted in treatment group, while no side effects were noted in the control group. There were 18 participants who dropped out of the study: three in control group due to non-compliance or loss to follow-up, and 15 in treatment group due to non-compliance, headache, or dermatitis rash.

McCallum et al. conducted a prospective comparative study in 2011 to evaluate the long-term benefits from topical NTG on LE [12]. The study followed the patients initially treated with topical NTG during initial RCT done by Paoloni et al. [10]. The aim of the study was to determine the long-term benefit of using topical NTG after cessation of therapy. The treatment group was given a topical NTG patch at 1.25mg/24h dose for six months, while the control group received a placebo patch. Both the groups also underwent a tendon rehabilitation programs. Study participants were assessed five years after discontinuation of treatment and included patient-rated pain scores, lateral epicondylar and proximal common extensor tendon tenderness, hand-held dynamometer measurement of resisted third finger metacarpophalangeal extension with a fully extended elbow (Maudsley’s test) and wrist extensor tendon mean peak force using a modified chair pick-up test (ORI-TETS). Of the 86 participants who were initially recruited for the RCT [10], 74 completed the six-month trial and 58 completed the five-year follow-up. The authors found that while both treatment and control groups demonstrated significant improvements in outcomes at baseline, topical NTG did not show any significant benefit over placebo after five years. The results from the study suggest no long-term benefits of topical NTG for LE after cessation of therapy compared to a standard tendon rehabilitation program alone. No side-effects were noted during the five-year follow-up.

Ozden et al. [13] did an RCT in 2014 to determine the effect of topical NTG for the short-term (three weeks) and long-term (six months) treatment of LE. The study included 40 participants with LE randomized into treatment and control groups, with treatment group receiving topical NTG patch (1.25mg/24 h) and control group receiving placebo patch. The patches were applied over the area of maximal tenderness once a day. Outcome measures included presence of pain, tenderness, and positive pain stimulating maneuvers using a VAS, with excellent or good results being considered successful. The study reported significant improvement in pain scores in the treatment group compared to control at three-week time point (3.15 in treatment group vs 6.45 in control group, p=0.001). There was also significant improvement in pain scores in the treatment group compared to the control group at 6 months (0.70 in treatment group vs 4.85 in control group, p=0.001). Two patients in the control group and one patient in the treatment group experienced headaches which resolved one week after study completion. There was no participant drop-out.

Discussion

LE, commonly known as tennis elbow, is an overuse tendinopathy originating from the forearm extensor tendons at the lateral humeral epicondyle. Our understanding of LE has changed over time, and it is no longer thought that tendinopathies are inflammatory in nature, therefore making traditional therapies including NSAIDs and corticosteroids less beneficial [1,2,4]. This systematic review evaluates the current evidence on the use of topical NTG for treatment of LE. The key findings from the studies included in this systematic review are described in Table 2. The current literature indicates a role for using topical NTG in treating LE, especially in the short-term and mid-term (up to six months). Topical NTG combined with participation in a tendon rehabilitation program significantly improves outcomes in patients with LE. The findings, although statistically significant, are likely to be clinically signifiant as well in real-world settings. The tendon rehabilitation programs used in the studies generally included rest from aggravating activities, early protective bracing, stretching, and a system of a gradual increase in exercise load. The outcomes described were decreased pain, increased function, and increased range of motion of the elbow. The underlying principle for improved outcomes in patients with LE is theorized to be secondary to increased collagen synthesis in the tendon which leads to development of a strong scar along appropriate lines of tension for the wrist extensors [2].

Only one study evaluated the long-term benefits of using topical NTG but found no significant outcomes in patients after five years of treatment. This study examined outcomes in patients five years after stopping the use the NTG, with initial treatment consisting of six-month use of topical NTG; however, the study did not include participants who were treated with topical NTG longer than six months. It is possible that for patients to experience long-term benefits of topical NTG for LE, they may need to be treated with NTG for a duration longer than six months. We also speculate that early and mid-term (up to six months) improved outcomes in patients treated with NTG would improve their elbow function earlier, thus improving their quality of life, and leading to an earlier return to baseline activities. Further robust studies are needed to determine the long-term effect of the use of topical NTG for treating LE. It should be noted that topical NTG does provide long-term benefits in treating Achilles tendonitis [12,14] and thus, would likely have similar effect for treating LE. In the meantime, clinicians must weigh the benefits of using NTG versus the side-effect risk of using longer-term NTG.

The current literature on the use of topical NTG for treating LE indicates a low side effect profile of NTG. The most common side effects found were headaches, hypotension, and dizziness, thought to be secondary to decrease in blood pressure due to use of NTG. It must be highlighted that the topical NTG dosage is less than what is typically used for the treatment of angina, which means lower side effects of NTG when used for LE as compared to its use for angina. Topical NTG also likely has a lower side-effect profile when used in the long-term compared to the use of corticosteroid injections. However, a standardized optimal treatment dose of topical NTG in the context of LE needs to be determined in future studies.

The limitations of the findings described in this systematic review include a low number of studies which have evaluated the effectiveness of topical NTG for treating LE. Out of the four studies included, three studies were done by the same research group which limits the validity of the findings across a wider setting. There is also no standard tendon rehabilitation program described which reduces the real-world applicability of these findings, although the current evidence does indicate improved outcomes in patients who underwent concurrent tendon rehabilitation instead of stretching-only exercises. Future studies are required to provide more robust data on the use of NTG for treating LE, including data on which components of concurrent tendon rehabilitation programs provide the most benefits. Future studies could also try to evaluate the effectiveness of using topical NTG in conjunction with short-term use of corticosteroid injections as well as other treatment modalities, despite treatment with corticosteroids having been shown to have high recurrence rates [4]. A clinical trial is currently in progress looking at sclerosing therapy using Polidocanol combined with extracorporeal focused shockwave therapy, painful daily eccentric training, along with daily topical nitric oxide for treatment of various tendinopathies including LE [15]. Lastly, another limitation of this systematic review is that we only included articles published in English, as it is possible that there may be studies published in other languages which were not included in this review. It is also worthwhile noting that although we did not include studies conducted on children in this systematic review, there could be a role of NTG in in treating LE especially in older children. However, before considering NTG in those cases, it will be important to consider osteomyelitis and Reiter's syndrome in children before making a clinical diagnosis of LE [16,17].

## Conclusions

In summary, this systematic review describes the use of topical NTG patches for treating LE in short- and mid-term pain along with elbow function. The most commonly used dose of NTG was a 1.25mg/24h transdermal patch. The treatment with NTG is non-invasive, low-cost and has a low side-effect profile. It can be used as a stand-alone or adjunct along with corticosteroids. It is important to note that a robust tendon rehabilitation program still plays a significant role in treating LE, although NTG does provide additional benefits in pain reduction and elbow function. Topical NTG may be an effective and safe treatment option that can be particularly used by emergency department physicians and even family doctors for treating LE among their adult patients, with potential implications for pediatric patients as well. However, more studies are required to fully understand the effect of topical NTG on LE, particularly the effective dose range, efficacy and safety in children along with long-term benefits.

## References

[1] Assendelft W, Green S, Buchbinder R, Struijs P, Smidt N (2003). Tennis elbow. BMJ.

[2] Khan KM, Cook JL, Kannus P, Maffulli N, Bonar SF (2002). Time to abandon the "tendinitis" myth. BMJ.

[3] Bisset L, Beller E, Jull G, Brooks P, Darnell R, Vicenzino B (2006). Mobilisation with movement and exercise, corticosteroid injection, or wait and see for tennis elbow: randomised trial. BMJ.

[4] Orchard J, Kountouris A (2011). The management of tennis elbow. BMJ.

[5] Murrell GAC, Szabo C, Hannafin JA (1997). Modulation of tendon healing by nitric oxide. Inflamm Res.

[6] Murrell GAC, Szabo C, Hannafin JA (2010). Evidence on the effectiveness of topical nitroglycerin in the treatment of tendinopathies: a systematic review and meta-analysis.. Archives Physical Med Rehab.

[7] Moher D, Liberati A, Tetzlaff J (2009). Preferred reporting items for systematic reviews and meta-analyses: the PRISMA Statement. Open Med.

[8] Page MJ, Moher D, Bossuyt PM (2021). PRISMA 2020 explanation and elaboration: updated guidance and exemplars for reporting systematic reviews. BMJ.

[9] Paoloni JA, Appleyard RC, Murrell GA (2004). The Orthopaedic Research Institute-tennis elbow testing system: a modified chair pick-up test-interrater and intrarater reliability testing and validity for monitoring lateral epicondylosis. J Shoulder Elbow Surg.

[10] Paoloni JA, Appleyard RC, Nelson J, Murrell GAC (2003). Topical nitric oxide application in the treatment of chronic extensor tendinosis at the elbow. Am J Sports Med.

[11] Paoloni JA, Murrell GC, Burch RM, Ang RY (2009). Randomised, double-blind, placebo-controlled clinical trial of a new topical glyceryl trinitrate patch for chronic lateral epicondylosis. Br J Sports Med.

[12] McCallum SD, Paoloni JA, Murrell GAC (2011). Five-year prospective comparison study of topical glyceryl trinitrate treatment of chronic lateral epicondylosis at the elbow. Br J Sports Med.

[13] Ozden R, Uruç V, Doğramaci Y, Kalaci A, Yengil E (2014). Management of tennis elbow with topical glyceryl trinitrate. Acta Orthop Traumatol Turc.

[14] Paoloni JA, Murrell GA (2007). Three-year followup study of topical glyceryl trinitrate treatment of chronic noninsertional achilles tendinopathy. Foot Ankle Int.

[15] (2022). TENDOSHOCK-2010 Combination Therapy for Athletic Tendinopathies (TENDOSHOCK). https://clinicaltrials.gov/ct2/show/study/NCT01185951?term=nitroglycerine&cond=Epicondylitis&draw=2&rank=2.

[16] Opara NU, Osuala EC, Nwagbara UI (2022). Management of Salter-Harris Type 1 fracture complicated with osteomyelitis in a sickle cell disease patient: a case report and review of literature. Medicines (Basel).

[17] Opara NU (2021). Osteomyelitis infection disguised as Reiter's syndrome in a child: a case report. Clin Case Rep.

